# Noninvasive Methods for Fault Detection and Isolation in Internal Combustion Engines Based on Chaos Analysis

**DOI:** 10.3390/s21206925

**Published:** 2021-10-19

**Authors:** Thyago L. de V. Lima, Abel C. L. Filho, Francisco A. Belo, Filipe V. Souto, Thaís C. B. Silva, Koje V. Mishina, Marcelo C. Rodrigues

**Affiliations:** 1 Postgraduate Program in Mechanical Engineering, Federal University of Paraíba (UFPB), João Pessoa 58051-900, PB, Brazil; abel@les.ufpb.br (A.C.L.F.); belo@les.ufpb.br (F.A.B.); celocr@ct.ufpb.br (M.C.R.); 2 Federal Institute of Paraiba (IFPB), Itabaiana 58360-000, PB, Brazil; 3 Department of Mechanical Engineering, Federal University of Paraíba (UFPB), João Pessoa 58051-900, PB, Brazil; koje@ct.ufpb.br; 4 Department of Electrical Engineering, Federal University of Paraíba (UFPB), João Pessoa 58051-900, PB, Brazil; filipe.souto@cear.ufpb.br (F.V.S.); thais.c.borges@cear.ufpb.br (T.C.B.S.)

**Keywords:** chaos analysis, fault diagnosis, internal combustion engines, misfire, sound analysis

## Abstract

The classic monitoring methods for detecting faults in automotive vehicles based on on-board diagnostics (OBD) are insufficient when diagnosing several mechanical failures. Other sensing techniques present drawbacks such as high invasiveness and limited physical range. The present work presents a fully noninvasive system for fault detection and isolation in internal combustion engines through sound signals processing. An acquisition system was developed, whose data are transmitted to a smartphone in which the signal is processed, and the user has access to the information. A study of the chaotic behavior of the vehicle was carried out, and the feasibility of using fractal dimensions as a tool to diagnose engine misfire and problems in the alternator belt was verified. An artificial neural network was used for fault classification using the fractal dimension data extracted from the sound of the engine. For comparison purposes, a strategy based on wavelet multiresolution analysis was also implemented. The proposed solution allows a diagnosis without having any contact with the vehicle, with low computational cost, without the need for installing sensors, and in real time. The system and method were validated through experimental tests, with a success rate of 99% for the faults under consideration.

## 1. Introduction

Global spending on car accidents is approximately USD 3.8 trillion a year, equivalent to Germany’s gross domestic product (GDP) [1], and they are associated with 1.25 million fatalities [2]. Some of these accidents are caused by mechanical failures. Even when fatalities do not occur, the breakdown of automotive vehicles can expose users to risky situations on the road.

Some of the vehicles in operation already have efficient electrical diagnostic systems, but regarding mechanical problems, corrective maintenance is still most commonly used. In addition, most drivers ignore problems in the operating conditions of their vehicles, not investigating their causes and therefore putting themselves at risk. This reality can be explained by the fact that car maintenance depends on the judgment of the technicians involved in the process, which results in a late diagnosis of the faults only after the vehicle breaks down [3].

Fault diagnosis systems in automotive vehicles have been researched and developed over the last three decades. However, 80% of countries market vehicles that fail to meet basic safety standards [2]. The literature highlights methods based on on-board diagnostics (OBD) [4,5], methods based on vibrational analysis [6], acoustic emissions [7], crankshaft speed measurements [8,9] and multiple sensors [10]. OBD-based systems, in most cases, do not provide information to identify the faulty component when detecting a failure [11]. In addition, OBD-based methods are dependent on the technology installed in vehicles. In turn, vibrational and acoustic emission-based methods have the disadvantage of performing only specific diagnoses, requiring multiple sensors for a broader approach.

As an alternative to the previously mentioned methods, techniques based on sound analysis are different in that they do not require direct contact with the monitored elements and are therefore considered totally noninvasive. However, the non-linearity of the automotive vehicle sound signal [12] and its complexity and difficulty to analyze [13] may be responsible for the little research in this area. In the automotive fault diagnosis literature, works can be found with the use of audio signal processing through the application of different techniques, such as wavelet decomposition [14], frequency separation filters [15], empirical mode decomposition, and sample entropy [12]. In addition, most research in the area deals with internal combustion engines [16,17].

The present work addresses the development of an integrated hardware and software platform for the detection and isolation of ignition (misfire) and belt failures that cause problems in the energy power generation system (EPGS). A physical device equipped with a microphone captures the audio signal emitted by the vehicle and transmits it to a smartphone, where the diagnosis is made. A computational algorithm for sound signal processing was developed using a chaos-based approach. The failure parameter adopted is the fractal dimension, used as the input of an artificial neural network (ANN) that is responsible for classifying the signals between normal and faulty. The proposed system and method are validated through experimental results. For comparison criteria, it adopted an approach based on the discrete wavelet transform, common in the literature and which presents good results when applied in fault diagnosis research [18,19].

The main contributions of this work to the state-of-the-art approach include the following: first, development of an embedded/portable system for the identification of misfire in a running engine with no contact; second, analysis and characterization of the sound of an internal combustion engine through chaos theory in which the fractal dimensions of the signal are used for the first time in the diagnosis of automotive vehicle failures, presenting a lower computational cost than techniques based on wavelets and analyses in the frequency domain; third, the system is inexpensive compared to benchtop equipment available on the market; fourth, a comparison of the results obtained with the application of the fractal dimension to the results obtained with the application of a more traditional method.

## 2. Classification of Chaotic Signals

### 2.1. Overview

A chaotic or nonlinear signal is characterized by its apparently random behavior, its broadband spectrum, and its high sensitivity to parametric perturbations and to the initial conditions [20]. Another important feature in the study of the time series obtained from the analysis of chaotic systems is that its fundamental nature is the determinism [21]. Although they originate from different physical phenomena, time series derived from chaotic systems have characteristics in common with those coming from stochastic processes, which makes them almost indistinguishable [22,23], namely, a broadband power spectrum, delta type auto correlation function, and unpredictable behavior overall.

Over the years, several methods of analysis have been developed for the detection of determinism in time series, such as techniques based on phase maps [24], algorithms based on entropy [25], algorithms based on nonlinear auto regressive models [26], methods based on the recurrence plot [27] and techniques based on the symbolic representation of the time series [28].

After checking for determinism, it becomes interesting to search for the main characteristic that ensures the existence of chaotic behavior, which is the sensitive dependence on the initial conditions [29]. One of the most important tests to verify the sensitivity to the initial conditions is the estimation of the largest Lyapunov exponent (LLE) [30]. Another method recently developed to identify the presence of chaotic behavior is the 0–1 test [31], which in comparison to the LLE, has the advantage of not requiring the reconstruction of the system’s phase space.

In addition to the LLE, other nonlinear measurements can also be used to estimate properties that describe nonlinear signals, such as the entropy, correlation, auto correlation and fractal dimensions [32]. The fractal dimensions (FD), as well as the LLE, are invariant cut-to-cut metrics in time series [33], which enables them to be applied in pattern recognition algorithms. Compared to other nonlinear methods that require a large amount of calculations, FD have a lower computational cost [34].

The FD value gives a quantitative measure of an object’s self-similarity [35], that is, how much a system is composed of smaller versions of itself. When dealing with time series, the FD reveals how many times a pattern is repeated in the time series [36]. FD have been employed in methods for diagnosing failures of rotating machinery [37] and rolling bearings [38], the analysis and classification of speech signals [39] and studies of natural phenomena [40]. In the chaos theory literature, several methods have been proposed for the calculation of FD, such as the methods of Higuchi [41], Katz [42] and Sevciks [43].

### 2.2. Verification of the Chaotic Behavior of the Vehicle’s Sound Signal

In the present work, the method proposed in [31] was chosen to combine the 0–1 test with a test for determinism. If the series fails the test for determinism, it can be concluded that it is stochastic or noisy, which compromises the application of the 0–1 test and consequently the use of tools such as FD.

**Figure 1:**
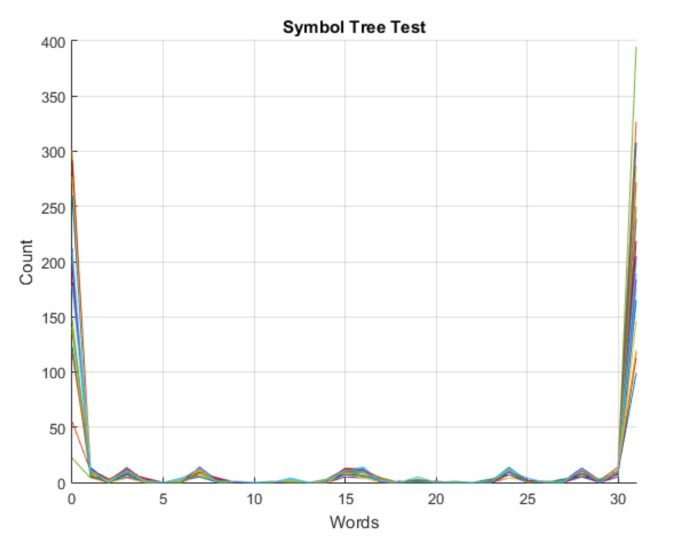
Result of the symbol tree test for the sound signal under study.

The adopted test for determinism was the symbol tree test [44]. Briefly, the method consists of the symbolic representation of the time series under consideration, partitioning the symbolic time series of length *N* into disjoint subsets of a given length *l* and then grouping the elements of each partition into “words” of a defined length *L*. The next step is the conversion of each word into base 10. Finally, the number of times each “word” in base 10 appears in the partition, it is plotted, generating the symbol spectrum of that partition. The graphical plotting of the symbol spectrum for each partition in the same graph reveals the nature of the time series: if it is deterministic, the symbol spectrum of each partition will be similar, with significant overlap, whereas in stochastic series, there will be little overlap from one spectrum to the next. Values of *N* = 10,000 samples, *l* = 500 and *L* = 5 were adopted and 20 spectra of symbols were generated, which are sufficient to determine the deterministic or stochastic nature of the time series, according to [31]. The result of the determinism test for the signal of the car in operation can be visualized in Figure 1, showing a significant overlap between the symbol spectra of the signal under analysis.

Next, the 0–1 test is implemented according to [31]. Given a time series with length N, by considering n ≪ N (n_cut_ = N/10), the modified mean square displacement is calculated as:

(1) $$M_{c}\left(n\right){=V(c)n+V}_{\mathrm{osc}}\left(c,n)+e(c,n\right)$$

where c is chosen randomly in the interval $\in \left(0,\pi \right)$, e(c,n) is an error term ($e\left(c,n\right)\to 0$ as n→∞). V_osc_ is given by:

(2) $$V_{\mathrm{osc}}\left(c,n\right)={\left(E\varphi \right)}^{2}\frac{1-\cos\mathrm{nc}}{1-\cos c}$$

The term Eφ is the mean error value of the time series. Subtracting the term V_osc_(c,n) from the mean square displacement, we obtain the modified mean square displacement:

(3) $$D_{c}\left(n\right){=M}_{c}\left(n\right){\;-\;V}_{\mathrm{osc}}\left(c,n\right)$$

**Figure 2:**
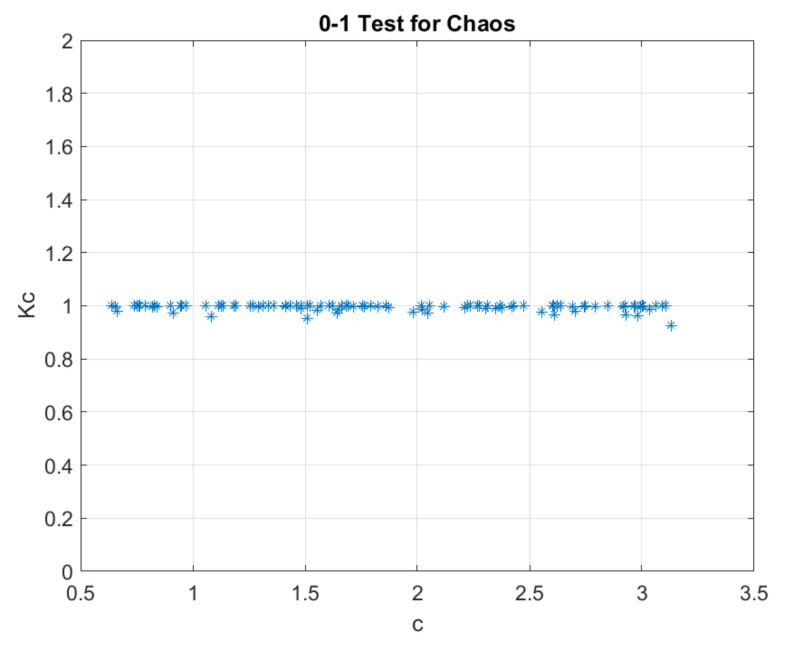
Result of the 0–1 test for chaos of the sound signal under study.

Finally, we find the asymptotic growth rate *K_c_* of the modified mean square displacement:

(4) $$K_{c}=corr\left(\xi ,\Delta \right)=\frac{\mathrm{cov}\left(\xi ,\Delta \right)}{\sqrt{\mathrm{var}\left(\xi \right)\mathrm{var}\left(\Delta \right)}}\in \left[-1,1\right]$$

where ξ = (1, 2, …, $n_{\mathrm{cut}}$) and Δ = ($D_{c}$(1), $D_{c}$(2), ..., $D_{c}$($n_{\mathrm{cut}}$). It is shown in [31] that the final value for characterizing chaotic behavior is the median of *K_c_* calculated for 100 different values of c, with *K_c_* = 1 for chaotic dynamics and *K_c_* = 0 for nonchaotic dynamics. Figure 2 shows the result of the 0–1 test for the audio signal acquired with the car in operation. The median for 100 values of *K_c_* was 0.9986, indicating the presence of a chaotic dynamic. 

This procedure was performed only to verify the chaotic nature of the signal and is not repeated in the fault diagnosis step. The adoption of this method ensures that the technique based on FD extraction can be used in an adequate manner, without risk of spurious results.

## 3. Wavelet Approach

### 3.1. Discrete Wavelet Transform

The discrete wavelet transform (DWT) was introduced in order to provide a more efficient description compared to the continuous wavelet transform (CWT). The DWT is not continuously translated or scaled but is translated or scaled at discrete intervals. There is the definition of DWT:

(5) DWTj,k=a0j-12∫-∞+∞x(t)ψ*t-kb0a0ja0jdt

where $\psi \left(t\right)$ is the mother wavelet and $\psi^{*}\left(t\right)$ is its complex conjugate. The parameters j and k are integers, a_0_ > 1 is a fixed expansion parameter, b_0_ is the fixed translation factor. In the literature, parameter values are generally adopted as a_0_ = 2 and b_0_ = 1 as they eliminate CWT redundancy, ensuring the invertibility and formation of an orthonormal base by daughter wavelets. Adopting such parameters in Equation (5), we have:

(6) DWTj,k=2-j2∫-∞+∞x(t)ψ*2-jt-kdt

In 1988, an algorithm was proposed for the implementation of the DWT, known as Mallat’s pyramidal algorithm or multiresolution analysis (MRA) [45]. In his work, Mallat demonstrated that a signal can be decomposed into two components, approximation and detail, as well as be reconstructed from them. The approximation can be interpreted as a low-pass filter, which contains low frequency information from the original signal, and the detail can be interpreted as a high-pass filter containing high frequency information from the same original signal.

**Figure 3:**
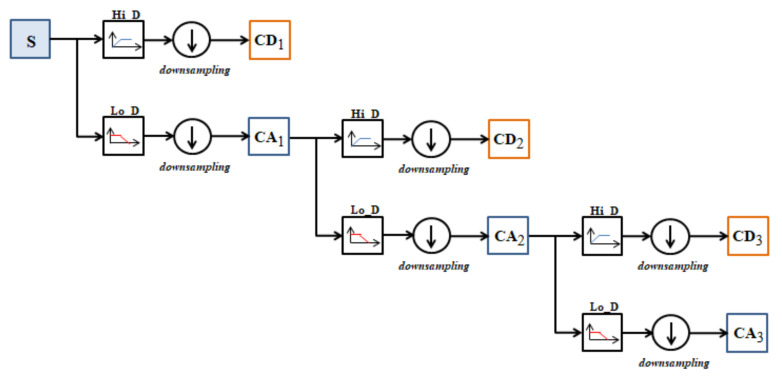
Illustration of a three-level decomposition of a signal.

The algorithm for MRA is divided into two parts, the decomposition and the reconstruction of the signal. The first step is to obtain vectors with the approximation and detail coefficients of the original signal in the decomposition step. Such vectors are obtained by convolution of the original signal with the low-pass filter (Lo_D_) for approximations and with the high-pass filter (Hi_D_) for details. Then, the operation called down-sampling is performed, which consists in eliminating the odd index values. The decomposition operation at three levels of a signal is illustrated in Figure 3.

The decomposition process can be iterated, with successive approximations being decomposed at a time, allowing the signal to be divided into many lower resolution components [46] The length of each filter is 2N, where N is the desired decomposition level. The length of the convolution vector is n+2N−1, where n is the signal length that will pass convolution with the filters. The coefficients CA_i_ (approximation coefficients at level i) and CD_i_ (detail coefficients at level i) have length $\left(\frac{n-1}{2}\right)+N$. After each convolution, the result will have approximately half the number of points of the vector in its previous state.

### 3.2. Wavelet Algorithm Validation

The algorithm for calculating the DWT was implemented in the Android environment. For evaluation criteria, a numerical calculation software was used as a comparison parameter, evaluating the algorithm behavior in situation of signal decomposition.

**Figure 4:**
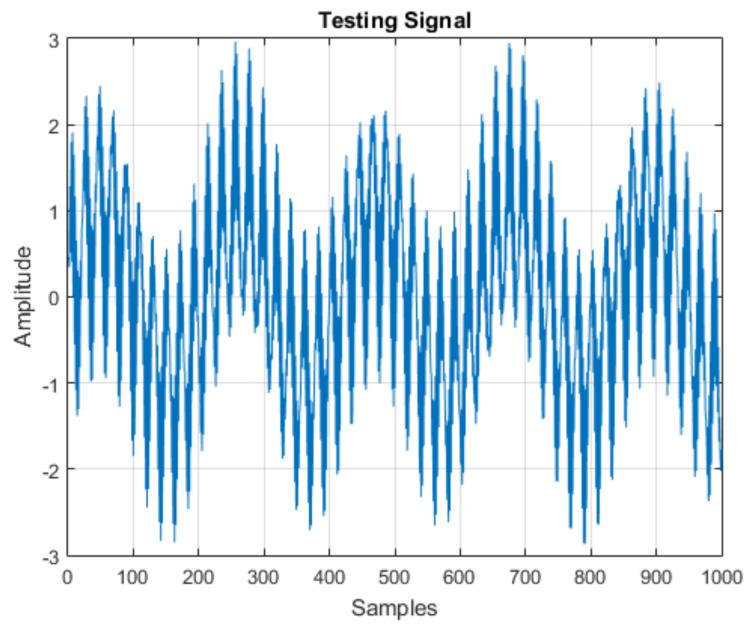
Test signal for MRA.

For evaluation, the signal was used, as shown in Figure 4. Initially, it was decomposed into one approximation and seven details, such that the coefficient vector was as follows:

(7) $$\left[{\mathrm{CA}}_{7}{\;\mathrm{CD}}_{7}{\;\mathrm{CD}}_{6}{\;\mathrm{CD}}_{5}{\;\mathrm{CD}}_{4}{\;\mathrm{CD}}_{3}{\;\mathrm{CD}}_{2}{\;\mathrm{CD}}_{1}\right]$$

**Figure 5:**
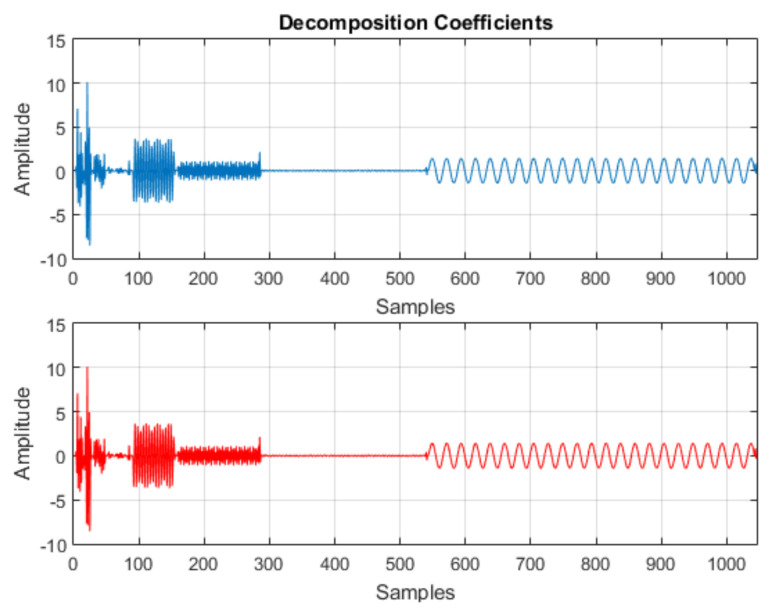
Wavelet decomposition coefficients—numerical calculation software (blue) and application (red).

Figure 5 shows the results obtained for signal decomposition in the numerical calculation software and in the application.

**Figure 6:**
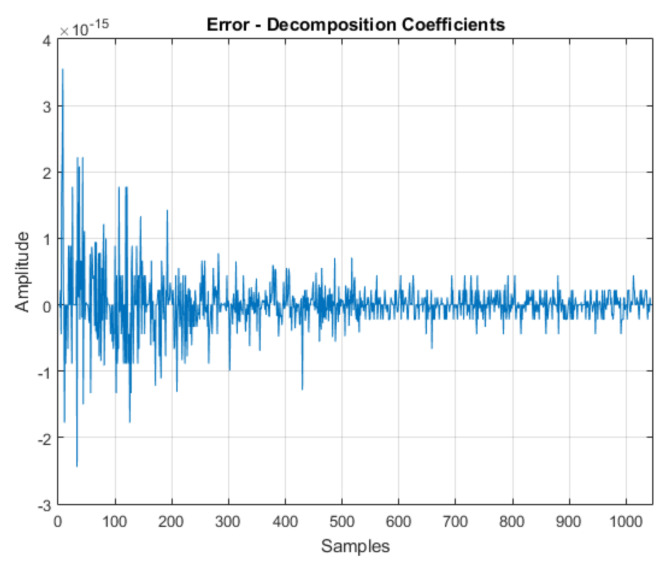
Error between the numerical calculation software and application results—wavelet decomposition.

The results obtained were compared, the error originating from the comparison of the results presented by the numerical calculation software and the application for this situation presented an average value of $1{.04\;\times \;10}^{-17}$, with a maximum value $3{.55\;\times 10}^{-15}$. The result of this comparison is seen in Figure 6. The results obtained in the application were considered adequate for the research objectives.

## 4. Methodology

### 4.1. Sound Signal Acquisition and Processing System

**Figure 7:**
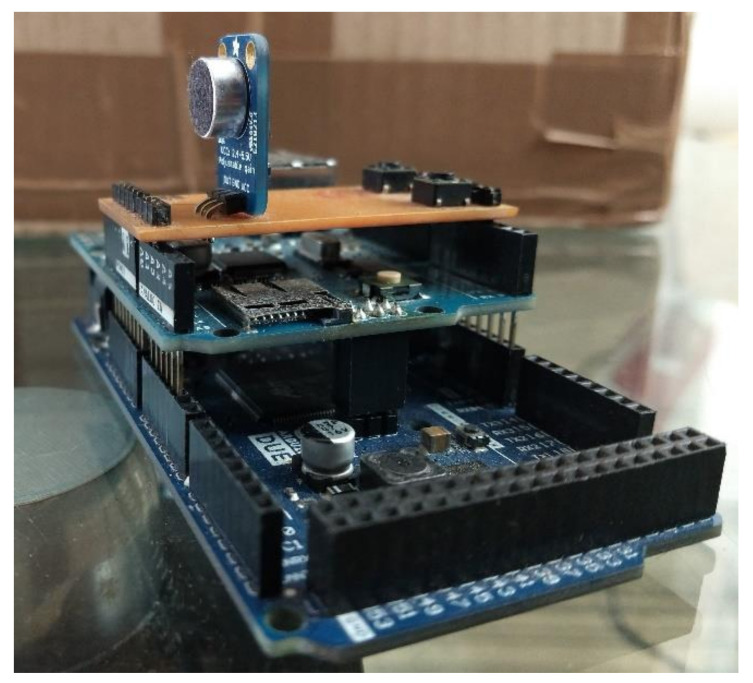
Sound acquisition system.

A low-cost system was devised, and for this reason, the price variable was also considered during the selection of components. As is usual in the recording industry, the system has an acquisition rate of 44.1 kHz, with a 16-bit resolution (ADC LTC1859). The format chosen for the audio file was WAVE, which guarantees uncompressed (lossless) audio storage. The system (Figure 7) essentially comprises the development platform, the microphone and the processing software. The development platform adopted was the Arduino Due, which, unlike the other most popular models of the company, has high computational capacities thanks to its SAM3X8E processor (manufactured by Microchip Technology, Chandler, AZ, USA), an ARM Cortex M3 processor. For the microphone selection, in addition to the price, the desired operating range (20 Hz up to 20 kHz) was considered. A breakout board with the CMA-4544PF-W electret condenser microphone (manufactured by CUI Devices, Lake Oswego, OR, USA) was chosen, which according to the frequency response curve provided by the manufacturer, is stable for the adopted operating range, and the MAX4466 operating amplifier (manufactured by Analog Devices, Norwood, MA, USA) was used for preamplification.

**Figure 8:**
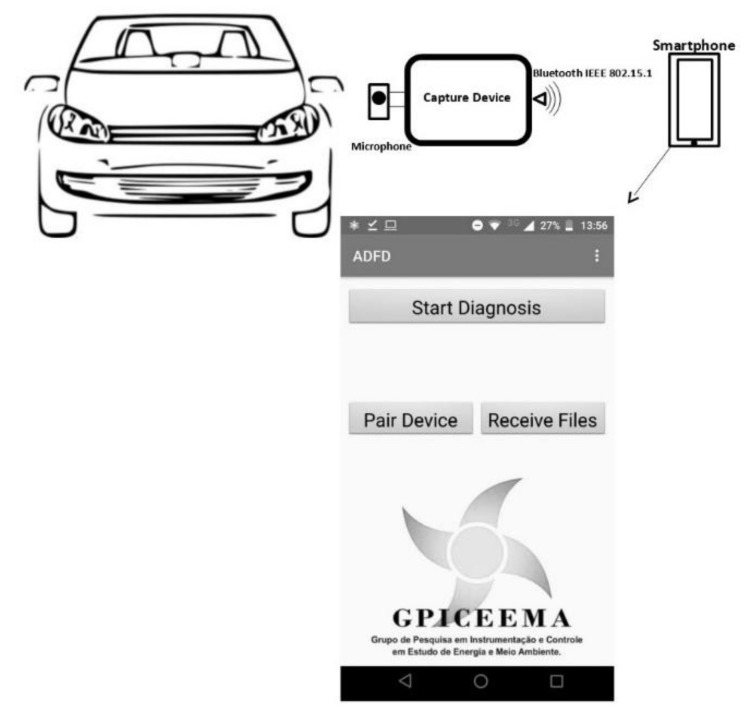
Illustration of the developed system’s application.

The implementation of the acquisition and processing system is shown in Figure 8. When triggered by the user, the sound emitted by the car is captured by the system and stored in WAVE format on a SD card. Then, a vector with the signal information is sent to the smartphone through a Bluetooth device HC05 (manufactured by HiLetgo, Shenzhen China. The data are then loaded, and the diagnostic routine is performed, making the result available on the smartphone screen. The processing routine was developed on Android Studio, which provides a development environment that encompasses and incorporates the IntelliJ IDEA IDE, the Android SDK (developed by Google LLC, Mountain View, CA, USA) plug-ins, and an emulator. The application was designed to run on any phone that has Android version 4.4 or higher.

It was decided not to carry out the sound signal acquisition process directly on the smartphone to avoid a limitation of the proposed application, since different smartphones have microphones with different responses in the reproduction of certain frequency components. In addition, experiments conducted during the study found that smartphone microphones do not show good sensitivity in the low frequency region.

### 4.2. Studied Faults

Faults in the belt, an integral component of the EPGS, and failures in the combustion process (misfire) were considered. Belt failures can lead to a loss of power, increased emissions and severe engine damage. In particular, its slip causes heat to be generated, which in turn migrates from the pulley to the shaft and rolling bearing and can cause the latter to fail prematurely. The alternator bearings and the cooling pump seals are especially sensitive to vibrations and heat [47]. Belt slip also causes the alternator output voltage to decrease [48], which reduces the battery life, as it will need to discharge more frequently to provide the additional power required by the load currents.

**Figure 9:**
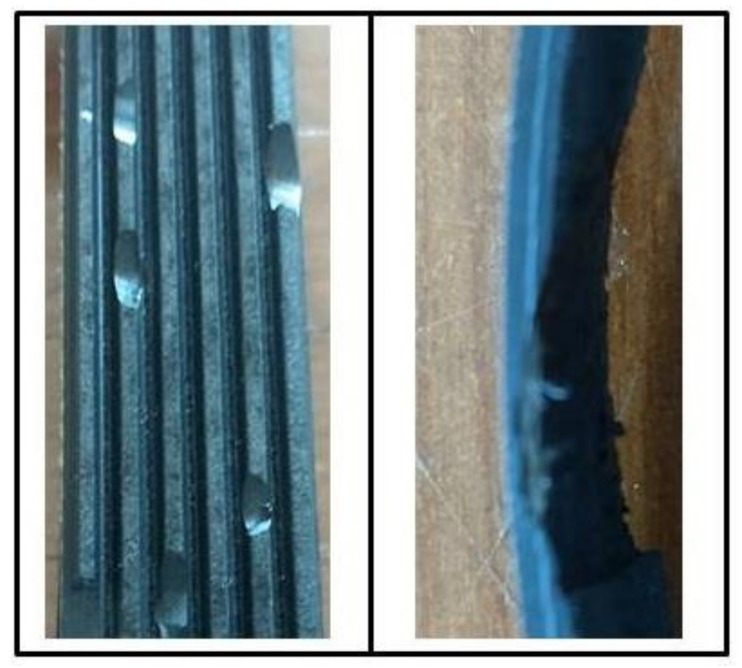
BPD fault (**left**) and BCL fault (**right**).

The faults considered for the belt were slip (BS), particle detachment (BPD) and concentrated loss of material (BCL). The BPD and BCL faults are represented in Figure 9. For the BPD fault, small parts of varying sizes were removed along the belt in all ribs. For the BCL fault, a single portion 25 mm in length and 2 mm in depth was removed.

Misfire is a fault that occurs when the fuel and air mixture cannot be burned in a single cylinder or in several cylinders of an internal combustion engine. Many reasons may result in engine ignition failure, such as a failure of the engine ignition system, a failure of the fuel injection system, a failure of the cylinder seals, etc. A misfire can result in decreased output power, increased fuel consumption, the discharge of excessive pollutants and even damage to the catalytic converter [49]. 

For the present work, misfire situations in one (SCM) and in two (DCM) cylinders were investigated. Failure was imposed by disconnecting the cables from the spark plugs corresponding to the cylinders.

### 4.3. Experimental Procedures

The vehicle used for the acquisitions was a Ford Fiesta 1.6 manufactured in 2006; its idle speed is 850 rpm +/− 50 rpm. The audio signals were acquired close to the vehicle exhaust pipe, adopting as standards a distance of approximately one meter and an angle of approximately 45° with respect to the vehicle, with a height of approximately 1.30 m. Although tests were initially carried out capturing the signal from the engine at the front, it was observed after data analysis that the option for the rear of the vehicle would have greater effectiveness in the proposed method. The acquisitions always occurred in the presence of a specialist. All signals were acquired with the engine in neutral, and the duration of all acquisitions was 5 s. The process was repeated in different environments and on different days. A total of 8 signal acquisition moments were performed in different scenarios within the university campus where the work was carried out: outdoors and indoors, with cold as well as warm engine conditions. There was an alternation between hours during the morning and afternoon. At times when acquisitions were made, occasional small background noises from activities carried out on campus by other people and from vehicles moving along nearby roads could be heard. The effect of these small background noises, as well as noises reflected in the physical obstacles close to carrying out the experiments, were not noticed after data processing. The smartphone used for the research was a Moto Z2 Play, with 4 GB of RAM and a Qualcomm Snapdragon 626 MSM8953 Pro processor, with 2.2 GHz (manufactured by Motorola, Schaumburg, IL, USA).

### 4.4. DWT Configuration

**Figure 10:**
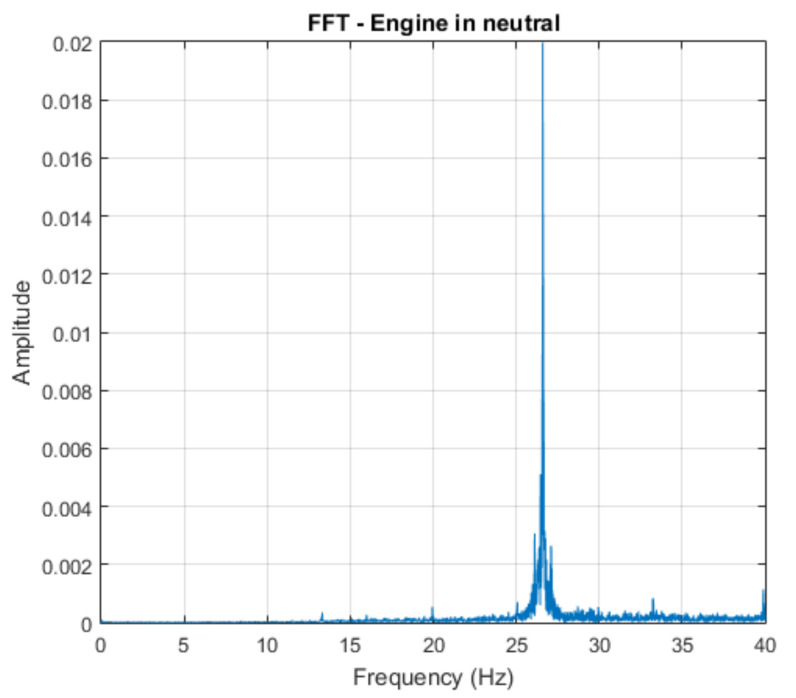
FFT of the engine in neutral and in normal conditions.

It is possible to establish a relationship between scale and frequency for each level of multiresolution analysis. It was decided to observe the detail that contains information related to the predominant frequency of the audio signal emitted by the vehicle engine running at neutral, in normal conditions. According to the FFT of the signal, exposed in Figure 10, the value is approximately 26.6 Hz. The number of decomposition levels was chosen to select the detail containing information related to the frequency of interest exposed. The decomposition level adopted was 10, according to the frequency band ranges shown in Table 1.

**Figure 11:**
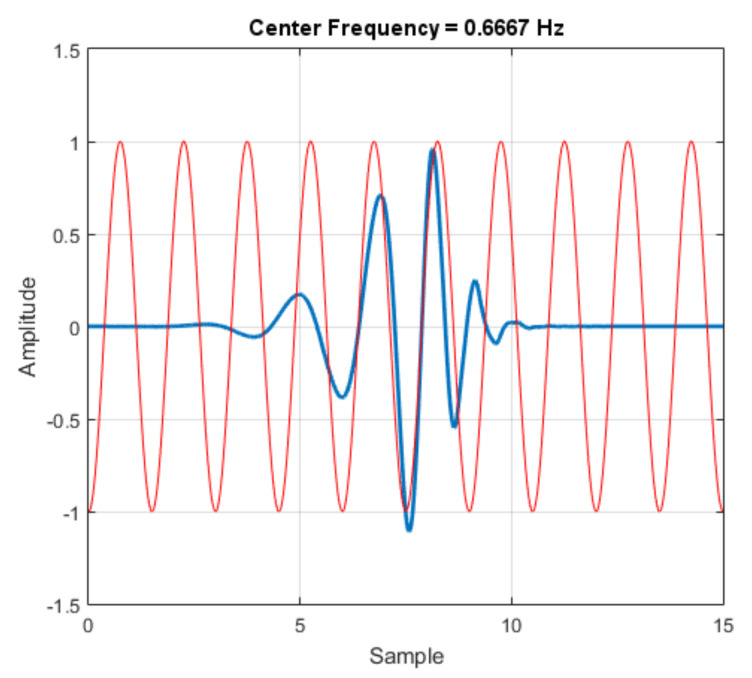
Wavelet (blue) and center frequency-based approximation.

According to [46], it is convenient to relate each of the scales to a known frequency sinusoid. Such a relationship is described in Equation (8) [50].

(8) $$F_{a}=\frac{F_{c}}{a\Delta }$$

where F_a_ is the frequency relative to the scale, or level, F_c_ is the center frequency of the chosen wavelet, a is the value of each scale per level, and Δ is the sampling period. The wavelet “db8” was adopted, whose center frequency is approximately 0.6667 Hz, as seen in Figure 11. With the values, the frequency relative to the scale is determined from Equation (8), resulting in a value of 28.71 Hz.

**Figure 12:**
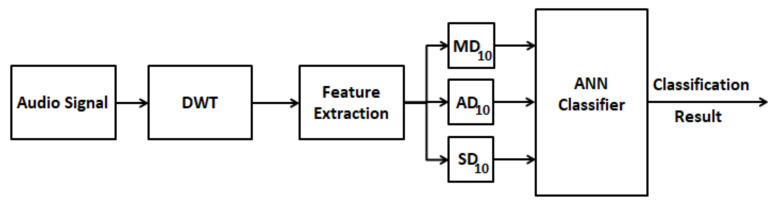
Schematic of the wavelet-based method for fault detection and isolation.

Finally, statistical parameters are calculated over the set of the wavelet coefficients. The features selected were: (a) mean of the absolute values of the coefficients in D_10_ (MD10), (b) average power of the coefficients in D_10_ (AD10) and (c) standard deviation of the coefficients in D_10_ (SD10). Features (a) and (b) provide information about the frequency distribution of the audio signal and feature (c) provides information about the amount of change of the frequency distribution [51]. A block diagram schematic of the wavelet-based method is shown in Figure 12.

### 4.5. Applied Fractal Dimension

The present work used Petrosian’s method [52], in their variations a, b and c, which consist of a binary representation of the time series prior to the calculation of the FD:

(a) Average method—the value of the binary representation is assigned 1 if the value of the time series sample is above the signal average and 0 otherwise;
(b) Modified zone method—the value of the binary representation is assigned a value of 1 if the value of the time series sample is outside the limits of the mean plus or minus the standard deviation and 0 otherwise;
(c) “Differential” method—the binary representation sample receives the value 1 if the difference between two consecutive samples of the time series is positive and 0 if it is negative.

**Figure 13:**
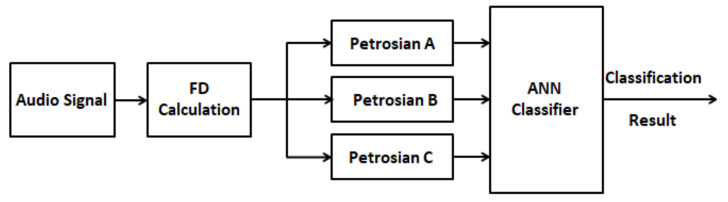
Schematic of the wavelet-based method for fault detection and isolation.

After performing the binary representations according to the methods previously described, the FD is then calculated as:

(9) $${\mathrm{FD}}_{\mathrm{Petrosian}}=\frac{\log\left(n\right)}{\log\left(n\right)+\log\left(\frac{n}{{n+0,4N}_{\Delta }}\right)}$$

where n is the signal length and N_Δ_ is the number of signal changes in the binary sequence. The schematic of the method based on fractal dimension for fault detection and isolation proposed is shown in Figure 13.

### 4.6. Classification Algorithm

Failure detection aims to recognize the abnormal behavior of components or processes through failures based on measured signals. Failure detection and diagnosis in general include three functions [53]:

(a) Fault detection: to indicate the presence of faults;
(b) Fault Isolation: to determine the location of faults after their detection;
(c) Identification of failures: to determine the degree of severity of failures and the time-varying behavior of failures.

For classification purposes, in the present work, a feed forward ANN with a supervised learning algorithm was applied, the back propagation. The network was trained using the descending gradient method, and the activation function adopted for the hidden layer and the output layer was a sigmoid function.

The ANN has a three-layer configuration, having as input the three parameters previously extracted, for both cases. The hidden layer presents 10 neurons and for the evaluation of the signals, the ANN presents 12 neurons in the output layer, with each fault represented according to Table 2. 

For the analyses, single failure situations and double/simultaneous failure situations, resulting from the combination of a misfire failure and a belt failure, were considered.

## 5. Results and Discussion

### 5.1. Acquisition System Tests

In order to analyze the quality of the signals acquired by the acquisition system, the following routine was adopted:

- Signals with known characteristics are emitted by a sound source and captured by the developed acquisition system;
- The captured audio is compared with the original signal to see if the main characteristics in the time domain are maintained;
- Finally, FFTs of the original signal and the recorded signal are performed, in order to observe whether the frequency domain characteristics are preserved;

The signals adopted for the analysis are described in Table 3.

For comparison purposes, the procedures described above are repeated with a commercial Sony Lcd Px-440 recording system. Acquisitions with the developed system and with the commercial recorder occurred simultaneously, keeping the same distance and positioning in relation to the source emitting the sound signal. 

**Figure 14:**
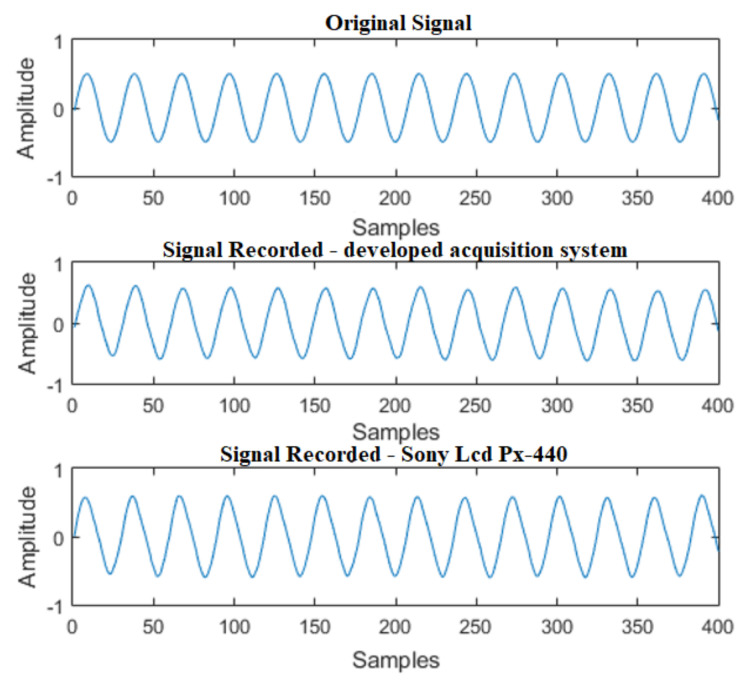
Acquisition of a single tone signal—1500 Hz sine wave.

In Figure 14, the signals for the single-tone test can be seen. It is verified that, in the time domain, the results of the developed system and of the commercial recorder are satisfactory, preserving the characteristics of the original signal. 

**Figure 15:**
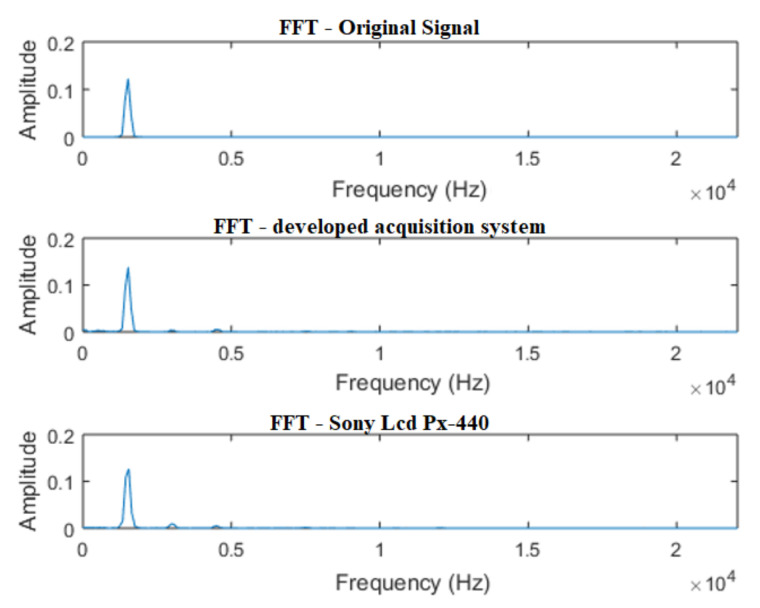
FFT of a single tone signal—1500 Hz sine wave.

In Figure 15, the FFT of the signals is properly demonstrated. It is noticed that both the signal acquired by the developed system, as well as the one acquired by the commercial recorder, adequately preserved the characteristics of the signal in the frequency domain.

**Figure 16:**
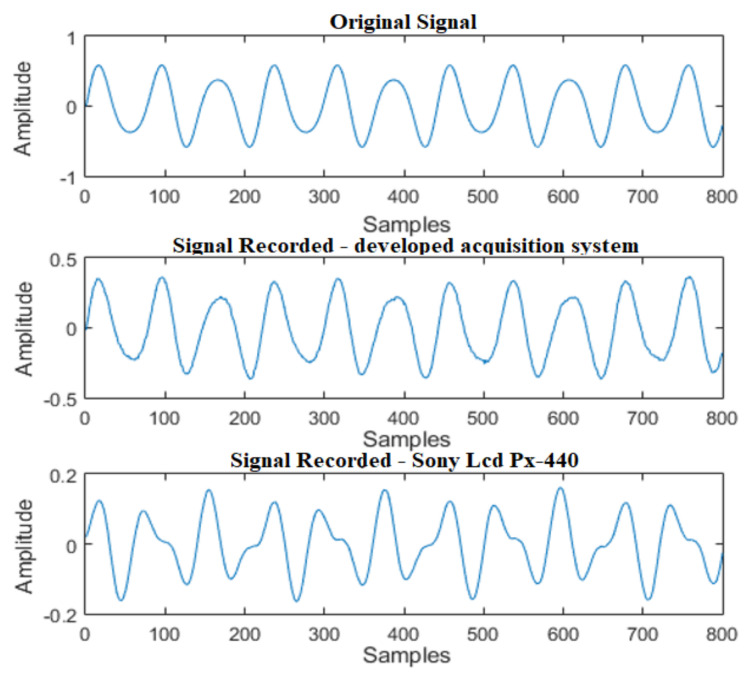
Acquisition of a two-tone signal: F1 = 600 Hz/F2 = 1000 Hz.

The results for the acquisition of the two-tone signal can be seen in Figure 16. It is observed that the developed acquisition system was able to acquire audio with characteristics close to the original signal. However, the commercial recorder used for comparison was not able to ensure a faithful reproduction of the emitted waveform.

**Figure 17:**
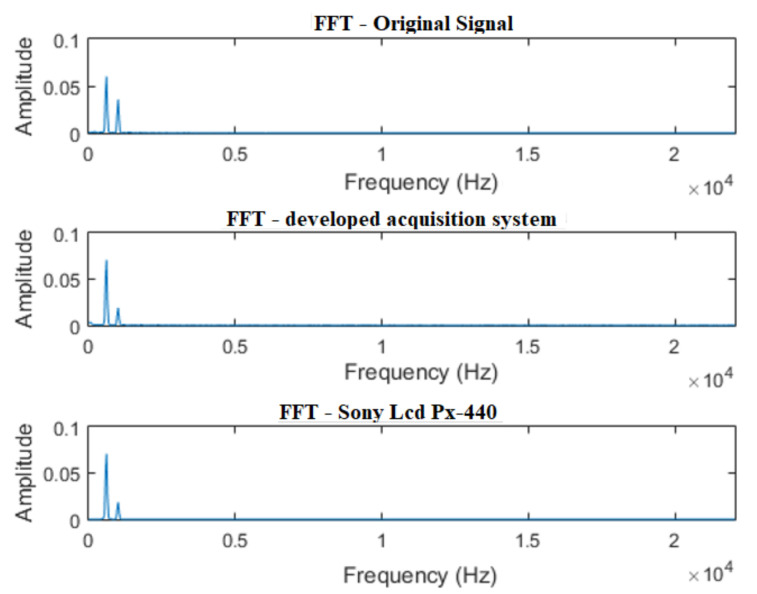
FFT of a two-tone signal: F1 = 600 Hz/F2 = 1000 Hz.

Regarding frequency domain analysis, the results of the two systems in comparison demonstrate that the peaks of the original signal were correctly identified in the signals acquired with the sound acquisition systems in comparison, as shown in Figure 17.

**Figure 18:**
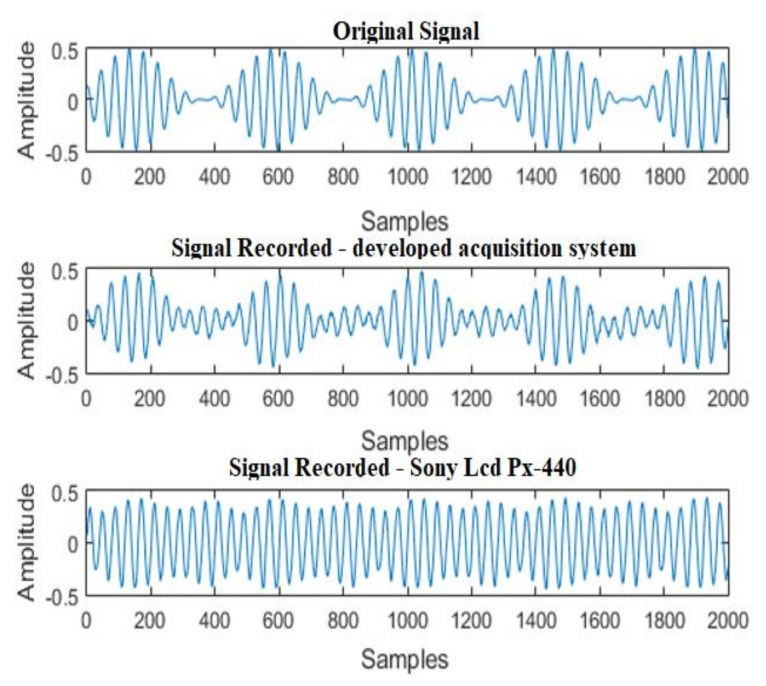
Acquisition of an AM signal—carrier: 1 kHz/modulator: 100 Hz.

In the last test performed, the results proved to be more discrepant. In Figure 18, it can be seen that the professional recording system failed to follow the behavior of the original AM signal. The developed system, however, managed to maintain the essence of the test signal, such behavior being considered satisfactory.

**Figure 19:**
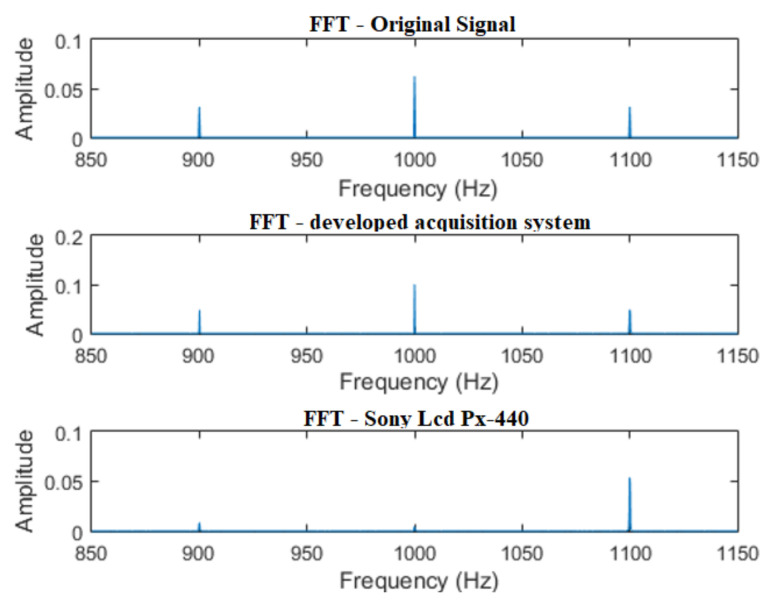
FFT of an AM signal—carrier: 1 kHz/modulator: 100 Hz.

In the frequency domain, as shown in Figure 19, the developed system was able to maintain the characteristics of the original signal. The commercial recorder, however, presented a performance below what was considered satisfactory.

According to the obtained results, it was considered that, in general, the developed sound acquisition system presents a performance in accordance with what is expected for the present research.

**Figure 20:**
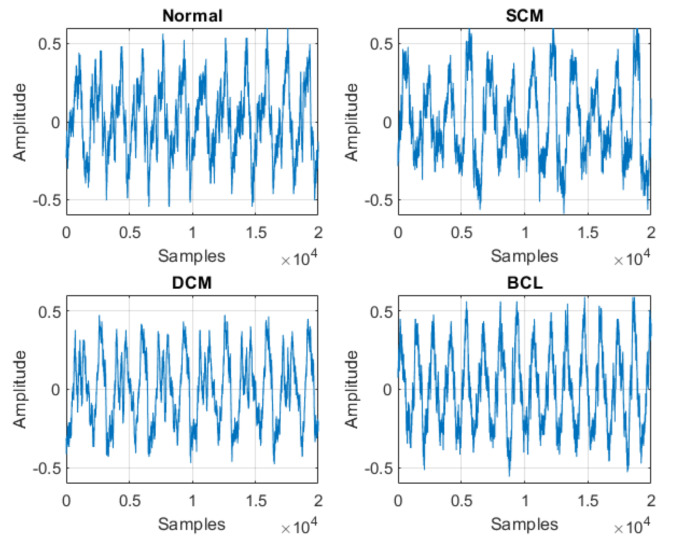
Samples of the studied signals—Normal, SCM, DCM and BCL.

**Figure 21:**
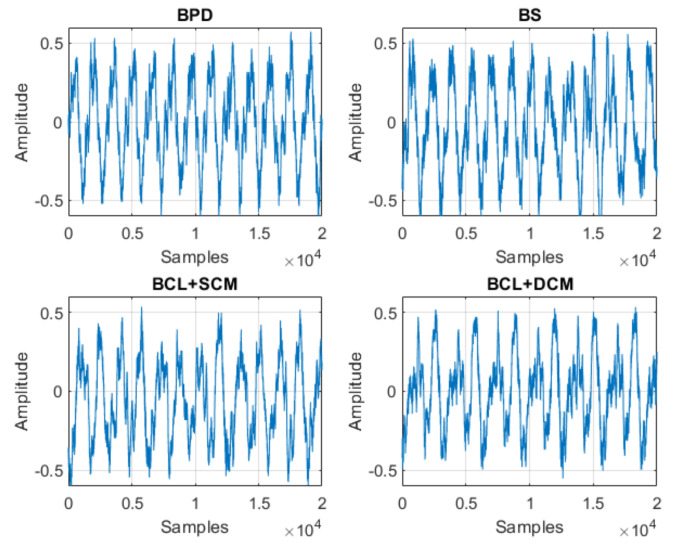
Samples of the studied signals—BPD, BS, BCL + SCM and BCL + DCM.

**Figure 22:**
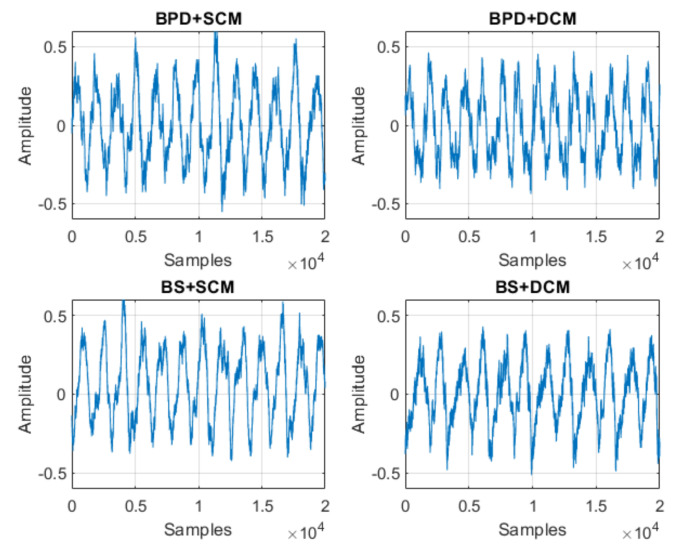
Samples of the studied signals—BPD + SCM, BPD + DCM, BS + SCM and BS + DCM.

With respect to the signals obtained, Figure 20, Figure 21 and Figure 22 represent samples of the raw signals obtained for the 12 classification states considered.

### 5.2. Wavelet-Based Fault Detection and Isolation Technique

The dataset used for classification included samples of the simple misfire and belt failures, as well as the combination of these two categories. For the SCM failure, for the absence of combustion in each one of the combustion chambers, 75 samples were collected, totaling 300 signals. For the DCM failure, absences of combustion in two chambers at a time were observed, considering the cylinders that operate in pairs in the combustion sequence. In this case, there were 150 samples for each pair, totaling 300 samples.

Considering the combined failures of the belt and misfire, the same logic was followed. For BCL + SCM, BPD + SCM and BS + SCM faults, the belt faults combined with the misfire fault in each one of the combustion chambers were considered. Thus, there were 75 samples for each chamber, totaling 300 samples for each one of these faults. For BCL + DCM, BPD + DCM and BS + DCM fault, 150 samples were considered for each pair of cylinders with no combustion, in combination with belt faults, caused simultaneously. Then, 3600 samples were used for the 12 categories of operation considered. 

Signal acquisitions lasted 5 s for each signal. Then, 2s were randomly extracted from each one of them to compose the set of 300 samples of each failure category. The training set consisted of 180 samples of each type of failure considered (2160 samples in total), randomly chosen. The remaining 120 samples from each failure category were then used in the classification step, totaling 1440 samples.

**Figure 23:**
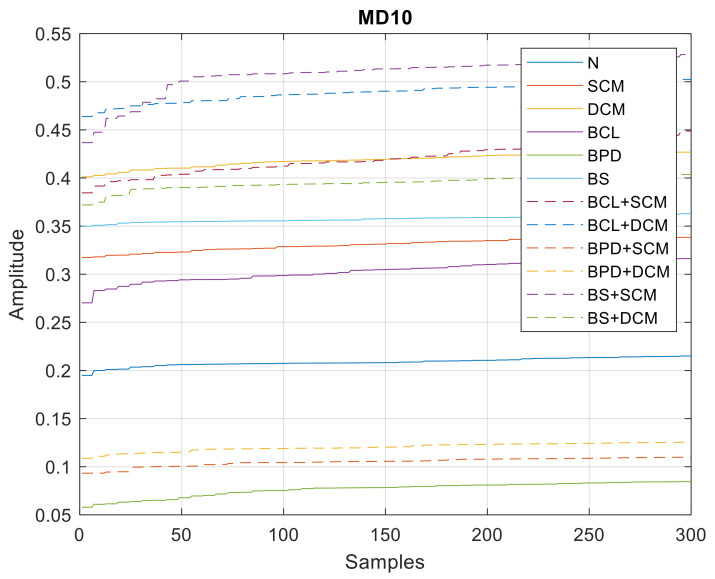
MD10 values—single and double/simultaneous faults.

**Figure 24:**
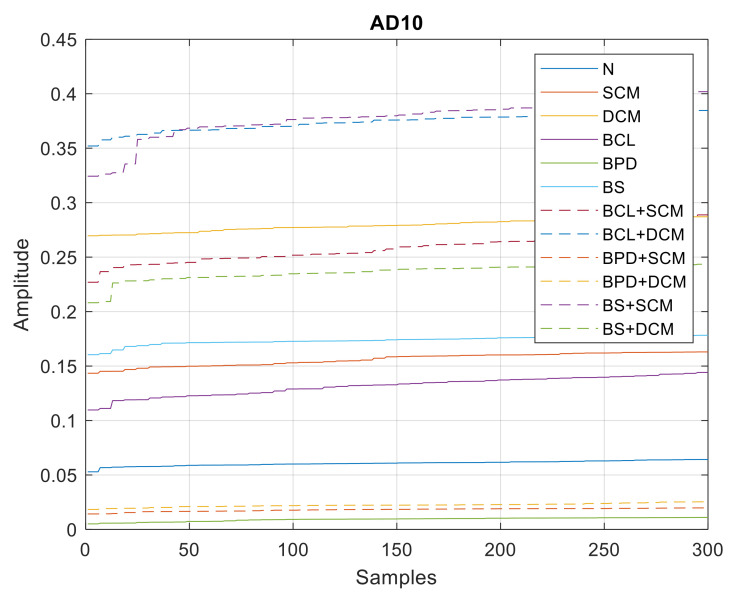
AD10 values—single and double/simultaneous faults.

**Figure 25:**
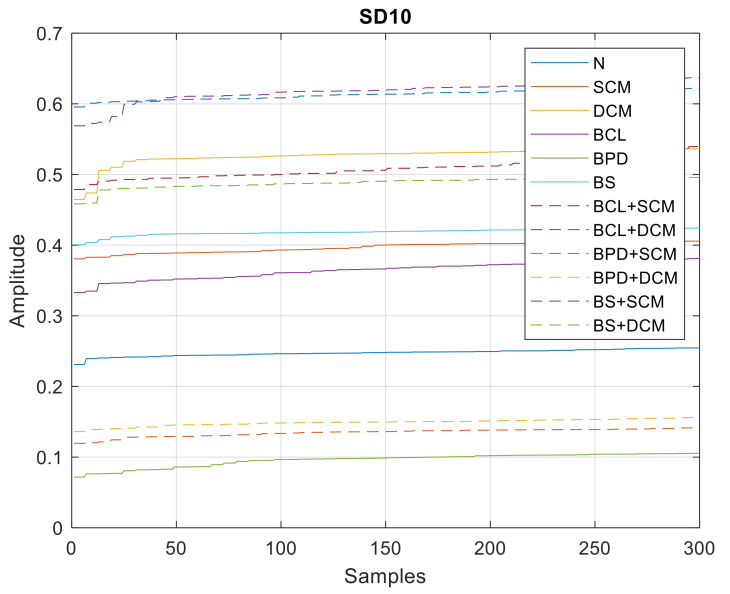
SD10 values—single and double/simultaneous faults.

In Figure 23, Figure 24 and Figure 25, all the values of the parameters of the wavelet MRA analysis are shown, whose minimum, average and maximum values are shown in Table 4.

**Figure 26:**
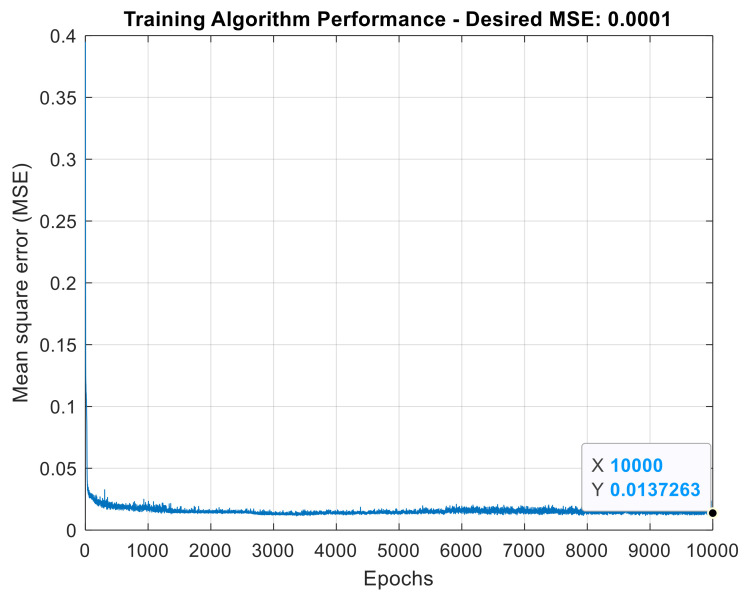
ANN training algorithm performance for wavelet AMR-based strategy.

The performance of the ANN in the training stage can be evaluated by looking at Figure 26, which plots the number of epochs required for convergence to the ANN mean square error target (MSE). In this case, the error target (0.0001) was not reached within the maximum number of epochs adopted (10,000).

Table 5 illustrates the confusion matrix for applying the MRA-based algorithm. The Precision column shows the percentages of all the examples predicted to belong to each class that are correctly classified. This metric is also called positive predictive value. The Recall row shows the percentages of all the examples belonging to each class that are correctly classified. This metric is also called true positive rate. The performance presented by the classifier was considered good, with an accuracy above 98%, presenting its worst performance for the BCL + SCM class (93.33% of recall).

### 5.3. Fault Detection and Isolation Technique Based on Fractal Dimensions

**Figure 27:**
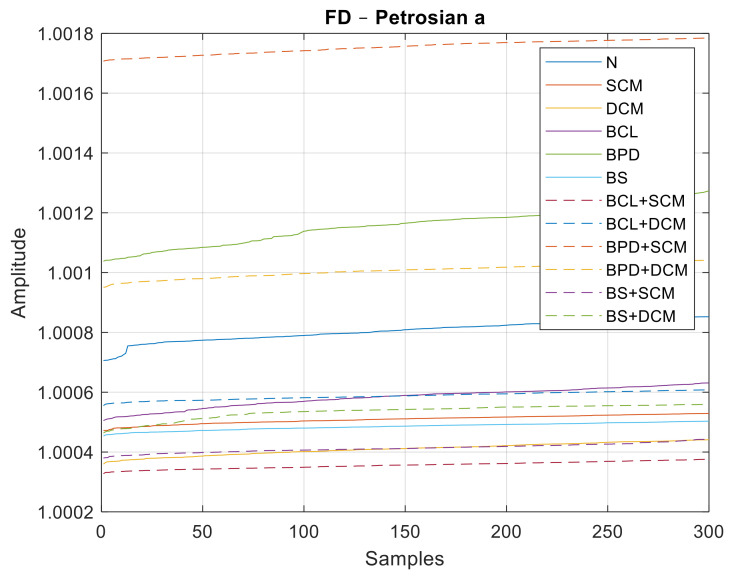
FD values for the Petrosian a method.

**Figure 28:**
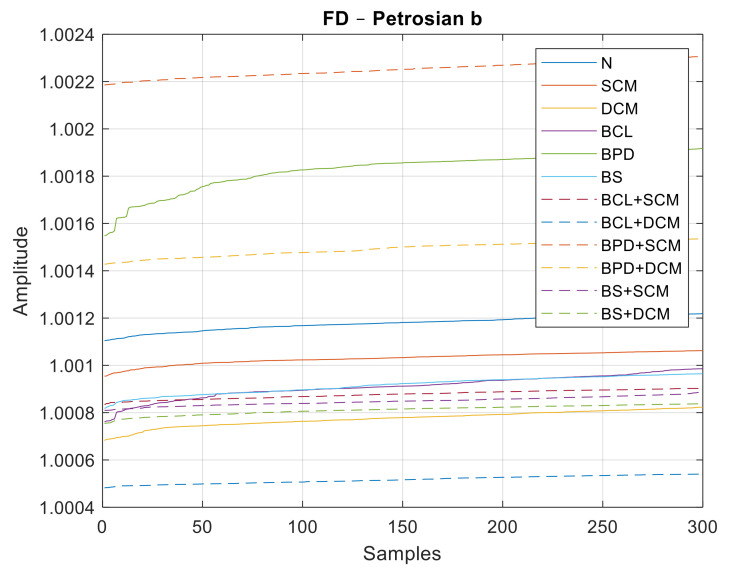
FD values for the Petrosian b method.

**Figure 29:**
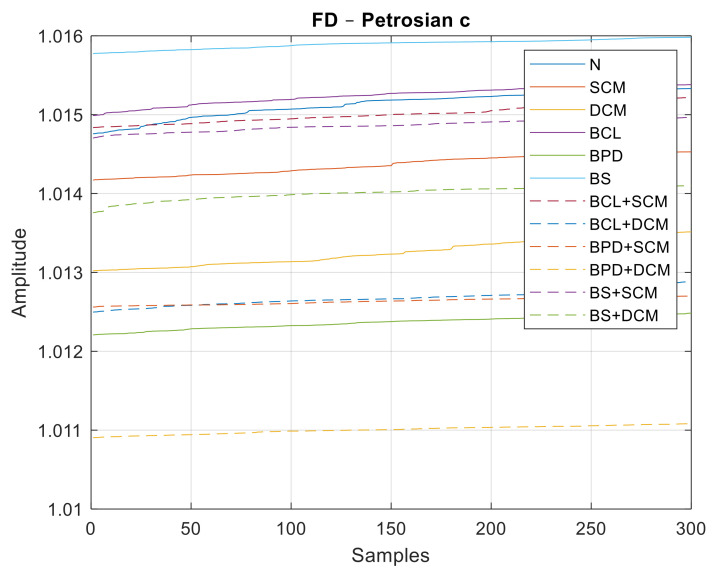
FD values for the Petrosian c method.

Similar to the previous case, data sets with 300 samples were used for each class of failure, 180 for training and another 120 for the classification step. In Figure 27, Figure 28 and Figure 29, all the values of the fractal dimensions extracted are shown.

**Figure 30:**
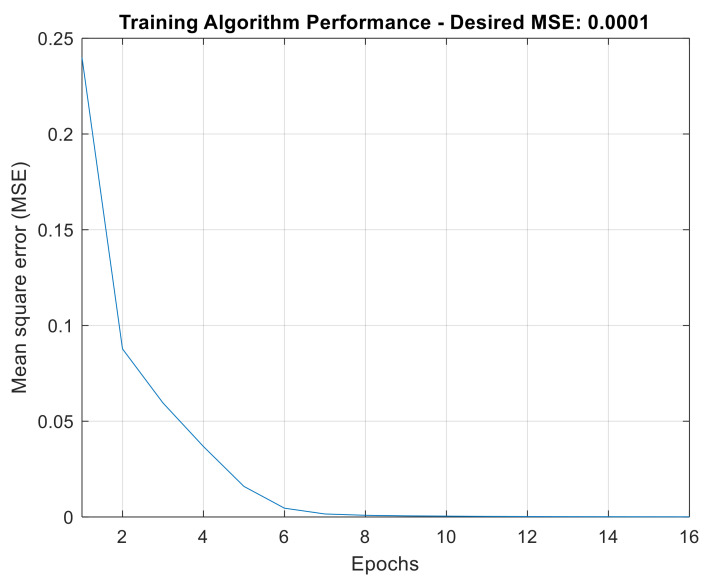
ANN training algorithm performance for FD-based strategy.

The performance of ANN in training is illustrated in Figure 30, which indicates a rapid convergence to the MSE, in only 16 epochs. The minimum, average and maximum values for the fractal dimensions extracted from the signals used are shown in Table 6. 

The confusion matrix (Table 7) reveals a good performance of the ANN in the classification task using fractal dimensions, with an accuracy of more than 99%. The worst case observed was for the BS + SCM class, whose recall rate was 95.83%.

### 5.4. Evaluation of the Application’s Overall Performance

The performances of applications based on the two proposed techniques were evaluated when considering their respective computational efforts. For this, the Android profiler tool was used, which summarizes the main information about the resources used by the application, such as memory allocation, CPU usage, energy consumption and bandwidth used in data transmission.

**Figure 31:**
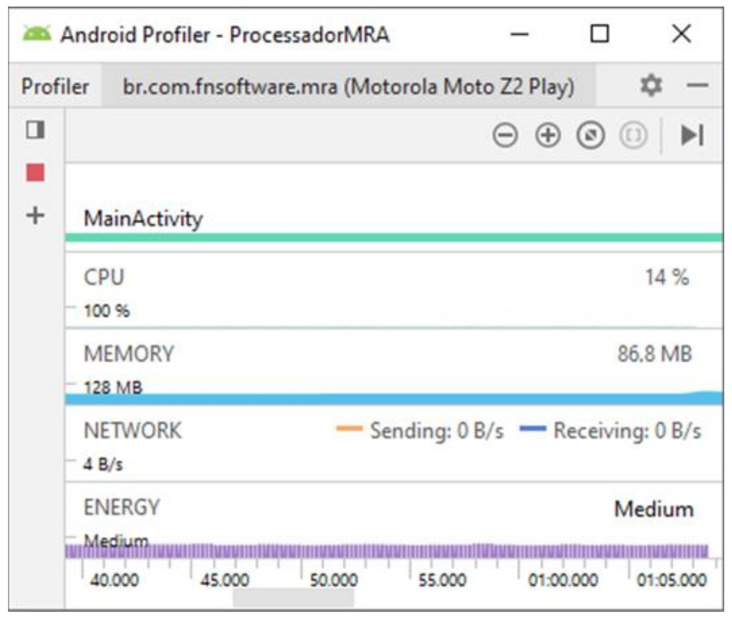
Overall app performance—wavelet MRA-based strategy.

**Figure 32:**
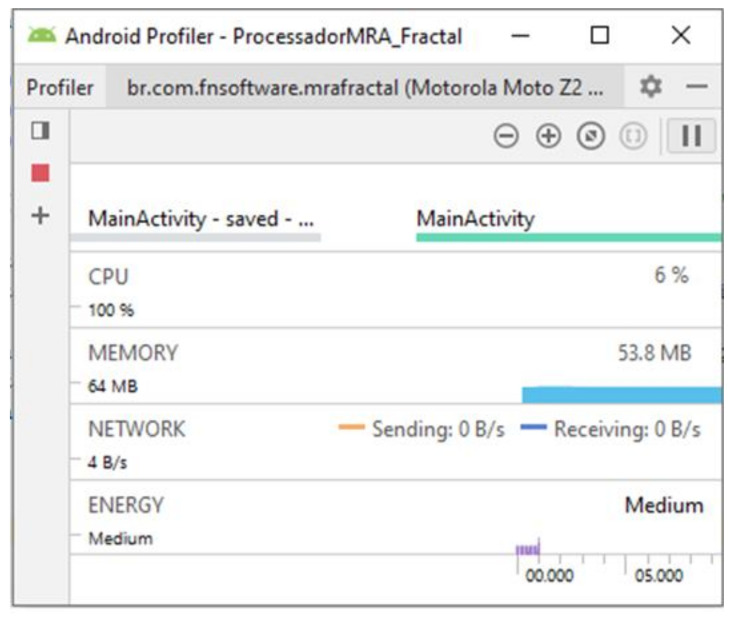
Overall app performance—FD-based strategy.

The results can be seen in Figure 31 and Figure 32. For the MRA wavelet analysis the processing time for a classification was approximately 68s. The initial memory allocation was 128 MB, with an average usage of 86.8 MB and a usage of 14% of the processor capacity. Application performance for the fractal dimension approach shows a significant decrease in computational effort and memory consumption, with a processing time of approximately 1 s. The initial memory allocation was 64 MB, with an average consumption of 53.8 MB. The application demanded 6% of the processing capacity. 

These tests were performed without other applications being opened by the Android device, with no internet connection and the device screen being kept turned on during the entire duration of the task execution.

From a general point of view, an accuracy of about 99% in the technique based on fractal dimensions developed in this work was obtained, which reveals a superior performance to similar works cited throughout the text [4,12,14,17]. From the point of view of the number of classes considered in the classification stage, the work also proved to be of some relevance, since 12 operating conditions were considered, a number lower than just one of the works mentioned. Furthermore, situations of simultaneous faults were considered, which according to [12] is a challenge due to the high cost of acquiring the signals and the almost always dependence of more than one type of signal in the considered system. The present work used only audio signals, captured by a single microphone in a low-cost system that was able to reflect the behavior of a wide region of the combustion engine. 

Another factor that becomes relevant is the fact that the classification process requires a low amount of signal features, only 3, in contrast to what can be seen in the works cited, which vary between 7 and 32. This fact may allow the inclusion of more features if necessary for the diagnosis of new types of faults, without compromising the computational effort criterion, since it is currently low. Still, from the point of view of computational effort, the technique based on fractal dimensions also presented a performance far superior to the wavelet, with lower execution time, memory allocation and demand for processing capacity. Particularly, the execution time of the technique for fractal dimensions around 1 s allows a diagnostic response time inferior to the classical monitoring techniques based on fast Fourier transform. 

As mentioned in the literature, it was experimentally verified that the FD are invariant cut-to-cut metrics in time series, as can be seen in Table 6, which made it possible to reflect the nature of the self-similarity of the signals, even in the presence of occasional noise background. Finally, the technique has a proven potential for application in embedded systems and mobile devices, given its nature of operating only performing the symbolic representation of the signal and extracting parameters based on non-complex mathematical operations.

## 6. Conclusions

The present study implemented a method for detecting and isolating faults in automotive vehicle combustion engines based on sound signal processing through the use of chaos theory and neural network techniques, and its comparison with an approach based on the wavelet MRA. In particular, misfire and belt faults were investigated, problems that can cause severe damage to the engine and place drivers at risk, involving them in situations of a complete operational breakdown or automobile accidents. As a differentiating feature, the work presented a solution that integrates low-cost hardware for the acquisition of signals with processing software running on smartphones with the Android operating system. The proposed strategy does not depend on the technology installed in the vehicle and is thus a solution with technological application potential for drivers and automotive workshops.

The method used showed satisfactory performance, reaching an overall accuracy of 99.58%. The application of fractal dimensions made the analysis fast and with a low demand for computational processing, unlike the technique that employ wavelets, which obtained similar performance compared to a more conventional approach. In addition, the adoption of sound signals means that the applied method is noninvasive, therefore requiring no intervention to the monitored vehicle for the installation of sensors.

## Figures and Tables

**Table 1:** Range of frequency bands in wavelet decomposition.

Decomposed Signal	Frequency Range (Hz)
D_1_	11.025–22.050
D_2_	5512.5–11.025
D_3_	2756.25–5512.5
D_4_	1378.12–2756.25
D_5_	689.06–1378.12
D_6_	344.53–689.06
D_7_	172.26–344.53
D_8_	86.13–172.26
D_9_	43.06–86.13
D_10_	21.53–43.06

**Table 2:** Representation of fault classes.

Neuron Outputs												
Condition	N1	N2	N3	N4	N5	N6	N7	N8	N9	N10	N11	N12
Normal (N)	1	0	0	0	0	0	0	0	0	0	0	0
SCM	0	1	0	0	0	0	0	0	0	0	0	0
DCM	0	0	1	0	0	0	0	0	0	0	0	0
BPD	0	0	0	1	0	0	0	0	0	0	0	0
BCL	0	0	0	0	1	0	0	0	0	0	0	0
BS	0	0	0	0	0	1	0	0	0	0	0	0
BCL + SCM	0	0	0	0	0	0	1	0	0	0	0	0
BCL + DCM	0	0	0	0	0	0	0	1	0	0	0	0
BPD + SCM	0	0	0	0	0	0	0	0	1	0	0	0
BPD + DCM	0	0	0	0	0	0	0	0	0	1	0	0
BS + SCM	0	0	0	0	0	0	0	0	0	0	1	0
BS + DCM	0	0	0	0	0	0	0	0	0	0	0	1

**Table 3:** Signals used for validation of the acquisition system.

Test Signal	Characteristic
Single tone—Sinusoidal	Fundamental Frequency = 1500 Hz
Two tones	F1 = 600 Hz/F2 = 1 kHz
AM signal	Carrier: 1 kHz/Modulator: 100 Hz

**Table 4:** Minimum, average and maximum values of the MRA parameters.

Parameters									
	MD10			SD10			AD10		
Conditions	Min	Med	Max	Min	Med	Max	Min	Med	Max
Normal (N)	0.19488	0.20876	0.21511	0.23106	0.24758	0.25454	0.05290	0.06073	0.06416
SCM	0.31622	0.33066	0.33831	0.38056	0.39690	0.40572	0.14341	0.15606	0.16299
DCM	0.40116	0.41845	0.42683	0.46469	0.52628	0.53641	0.26960	0.27919	0.28705
BPD	0.05792	0.07651	0.08451	0.07176	0.09593	0.10540	0.00510	0.00922	0.01101
BCL	0.27026	0.30302	0.31749	0.33278	0.36431	0.38114	0.10968	0.13159	0.14413
BS	0.35006	0.35731	0.36290	0.40071	0.41851	0.42416	0.16042	0.17358	0.17824
BCL + SCM	0.38449	0.42022	0.44873	0.47875	0.50758	0.53949	0.22694	0.25828	0.28879
BCL + DCM	0.46379	0.48876	0.50245	0.59556	0.61262	0.62177	0.35201	0.37393	0.38459
BPD + SCM	0.09333	0.10495	0.10989	0.11952	0.13464	0.14136	0.01423	0.01804	0.01979
BPD + DCM	0.10878	0.12028	0.12551	0.13605	0.14904	0.15606	0.01833	0.02230	0.02537
BS + SCM	0.43666	0.50717	0.52827	0.56887	0.61714	0.63686	0.32429	0.37767	0.40188
BS + DCM	0.37198	0.39480	0.40360	0.45845	0.48814	0.49585	0.20812	0.23606	0.24349

**Table 5:** Confusion Matrix—wavelet MRA.

Predicted Class	Target Class												Precision (%)
	N	SCM	DCM	BPD	BCL	BS	BCL + SCM	BCL + DCM	BPD + SCM	BPD + DCM	BS + SCM	BS + DCM	
Normal (N)	120	0	0	0	0	0	0	0	0	0	0	0	100
SCM	0	117	0	0	1	0	0	0	0	0	0	0	99.15
DCM	0	0	120	0	0	0	0	0	0	0	0	0	100
BPD	0	0	0	120	0	0	0	0	0	0	0	0	100
BCL	0	3	0	0	119	0	0	0	0	0	0	0	97.54
BS	0	0	0	0	0	120	0	0	0	0	0	0	100
BCL + SCM	0	0	0	0	0	0	112	0	0	0	0	2	98.24
BCL + DCM	0	0	0	0	0	0	0	120	0	0	4	0	96.77
BPD + SCM	0	0	0	0	0	0	0	0	120	5	0	0	96
BPD + DCM	0	0	0	0	0	0	0	0	0	115	0	0	100
BS + SCM	0	0	0	0	0	0	0	0	0	0	116	0	100
BS + DCM	0	0	0	0	0	0	8	0	0	0	0	118	93.65
Recall (%)	100	97.50	100	100	99.16	100	93.33	100	100	95.83	96.66	98.33	
	**Accuracy (%)**												98.40

**Table 6:** Minimum, average and maximum values of the FD extracted.

Parameters									
	FD—Petrosian a			FD—Petrosian b			FD—Petrosian c		
Conditions	Min	Med	Max	Min	Med	Max	Min	Med	Max
Normal (N)	1.00071	1.00080	1.00085	1.00110	1.00118	1.00122	1.01476	1.01513	1.01533
SCM	1.00047	1.00051	1.00053	1.00095	1.00103	1.00106	1.01417	1.01436	1.01453
DCM	1.00036	1.00041	1.00044	1.00068	1.00077	1.00082	1.01302	1.01325	1.01351
BPD	1.00103	1.00116	1.00127	1.00154	1.00183	1.00192	1.01221	1.01236	1.01248
BCL	1.00050	1.00058	1.00063	1.00076	1.00091	1.00099	1.01499	1.01524	1.01538
BS	1.00045	1.00049	1.00050	1.00082	1.00091	1.00096	1.01577	1.01589	1.01598
BCL + SCM	1.00032	1.00035	1.00038	1.00083	1.00088	1.00090	1.01483	1.01501	1.01522
BCL + DCM	1.00055	1.00059	1.00061	1.00048	1.00052	1.00054	1.01249	1.01268	1.01288
BPD + SCM	1.00171	1.00175	1.00178	1.00219	1.00225	1.00230	1.01256	1.01263	1.01270
BPD + DCM	1.00095	1.00100	1.00104	1.00143	1.00149	1.00153	1.01090	1.01100	1.01108
BS + SCM	1.00037	1.00041	1.00044	1.00080	1.00085	1.00089	1.01470	1.01486	1.01497
BS + DCM	1.00046	1.00053	1.00056	1.00075	1.00081	1.00083	1.01376	1.01401	1.01410

**Table 7:** Confusion matrix—FD strategy.

Predicted Class	Target Class												Precision (%)
	N	SCM	DCM	BPD	BCL	BS	BCL + SCM	BCL + DCM	BPD + SCM	BPD + DCM	BS + SCM	BS + DCM	
Normal (N)	120	0	0	0	0	0	0	0	0	0	0	0	100
SCM	0	118	0	0	3	0	0	0	0	0	0	0	97.52
DCM	0	0	120	0	0	0	0	0	0	0	0	0	100
BPD	0	0	0	120	0	0	0	0	0	0	0	0	100
BCL	0	1	0	0	117	0	0	0	0	0	0	0	99.15
BS	0	1	0	0	0	120	0	0	0	0	0	0	99.17
BCL + SCM	0	0	0	0	0	0	118	0	0	0	5	0	95.93
BCL + DCM	0	0	0	0	0	0	0	120	0	0	0	0	100
BPD + SCM	0	0	0	0	0	0	0	0	120	0	0	0	100
BPD + DCM	0	0	0	0	0	0	0	0	0	120	0	0	100
BS + SCM	0	0	0	0	0	0	2	0	0	0	115	0	98.29
BS + DCM	0	0	0	0	0	0	0	0	0	0	0	120	100
Recall (%)	100	98.33	100	100	97.50	100	98.33	100	100	100	95.83	100	
	**Accuracy (%)**												99.16

## Data Availability

Raw data are available from authors upon request.
